# Influence of the physico-chemical bioink composition on the printability and cell biological properties in 3D-bioprinting of a liver tumor cell line

**DOI:** 10.3389/fbioe.2023.1093101

**Published:** 2023-02-23

**Authors:** Anna Fritschen, Mariana Acedo Mestre, Sebastian Scholpp, Andreas Blaeser

**Affiliations:** ^1^ BioMedical Printing Technology, Department of Mechanical Engineering, Technical University of Darmstadt, Germany; ^2^ Centre for Synthetic Biology, Technical University of Darmstadt, Germany

**Keywords:** biofabrication, bioprinting, drop-on-demand (DOD), bioink development, cancer, liver, biomaterials, HepG2

## Abstract

The selection of a suitable matrix material is crucial for the development of functional, biomimetic tissue and organ models. When these tissue models are fabricated with 3D-bioprinting technology, the requirements do not only include the biological functionality and physico-chemical properties, but also the printability. In our work, we therefore present a detailed study of seven different bioinks with the focus on a functional liver carcinoma model. Agarose, gelatin, collagen and their blends were selected as materials based on their benefits for 3D cell culture and Drop-on-Demand (DoD) bioprinting. The formulations were characterized for their mechanical (G’ of 10–350 Pa) and rheological (viscosity 2–200 Pa*s) properties as well as albumin diffusivity (8–50 μm^2^/s). The cellular behavior was exemplarily shown for HepG2 cells by monitoring viability, proliferation and morphology over 14 days, while the printability on a microvalve DoD printer was evaluated by drop volume monitoring in flight (100–250 nl), camera imaging of the wetting behavior and microscopy of the effective drop diameter (700 µm and more). We did not observe negative effects on cell viability or proliferation, which is due to the very low shear stresses inside the nozzle (200–500 Pa). With our method, we could identify the strengths and weaknesses of each material, resulting in a material portfolio. By specifically selecting certain materials or blends, cell migration and possible interaction with other cells can be directed as indicated by the results of our cellular experiments.

## 1 Introduction

In recent years, three-dimensional biomimetic tissue models have become of greater interest in pharmaceutical research to compensate for late fails in clinical trials and to improve the efficiency of medication (Breslin and O’Driscoll, 2013; Terrell et al., 2020). To further increase reproducibility and fabrication speed, 3D-bioprinting has emerged as a technology capable of producing complex geometries, multi-cellular and spatially arranged tissue samples (Unagolla and Jayasuriya, 2019; Matai et al., 2020). Bioprinting has the potential to further accelerate automation and possibilities for in-line process control (Lindner and Blaeser, 2022).

**FIGURE 1:**
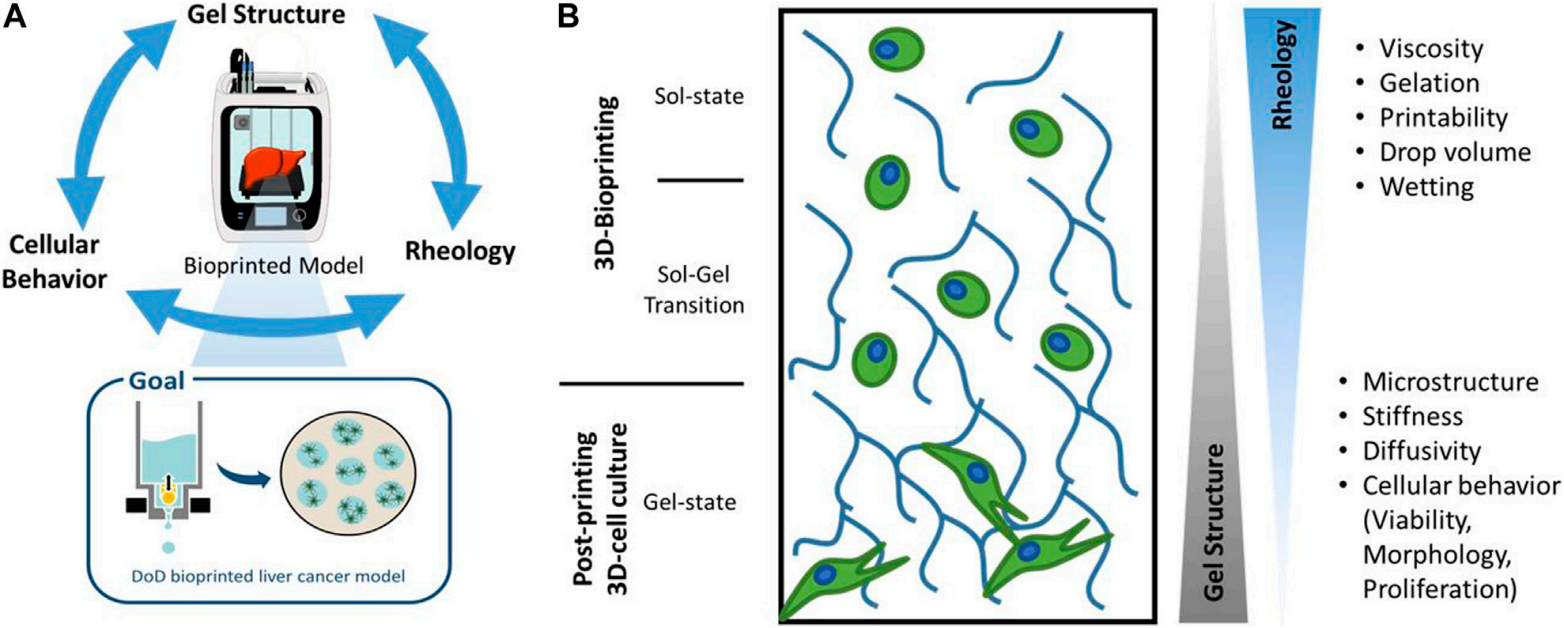
Schematic drawing of hydrogel properties and their influence on a Drop-on-Demand (DoD)-bioprinted liver cancer model. Different hydrogel properties dominate during the 3D-bioprinting process and in the subsequent 3D-cell culture, which are characterized in this work.

When considering hydrogels for use in bioprinting, various and at times opposing properties of the material play a crucial role in the future performance of the bioprinted tissue model (Patterson et al., 2010; Unagolla and Jayasuriya, 2019). The exact material requirements are governed by the planned tissue geometry, possible co-cultures and read-outs, with no single, outstanding hydrogel for all purposes. The microstructure, the rheology and the chemical composition influence cell functions, such as viability, proliferation, and gene expression, but at the same time influence the printability and post-printing stability (Parak et al., 2019) (Figure 1). By thoroughly characterizing and selecting the gel structure and chemical composition of the biomaterial, the cellular behavior inside the gels can be manipulated (Malda et al., 2013; Unagolla and Jayasuriya, 2019). This enables for example the fabrication of single cell models where a migration-hindering biomaterial plays a protective and binding role. Interactive models can be fabricated using a highly porous material offering cell adhesion sites for the study of spheroid formation, vascularization or cell migration.

Many times, contradictions between the biofunctionality and printability of a bioink are apparent. Extracellular matrix proteins such as collagen and fibrin offer an attractive environment for cells, as they allow cell adhesion over integrin-binding sites. However, their slow gelation kinetics, low mechanical stability and complex handling reduces the printing resolution and limits geometric complexity. On the other hand, plant-based materials such as agarose and alginate, offer superior printing properties, long-term stability in cell culture, affordability and ease-of-use, but lack cell adhesion motifs and often lead to changes in cell morphology, proliferation or vitality (Fritschen and Blaeser, 2020).

In this work, we studied agarose, gelatin and collagen as well as blends thereof, as they have shown good printability with microvalve based Drop-on-Demand printing systems (Köpf et al., 2016; Kreimendahl et al., 2017; Campos et al., 2019a), can be fabricated at viscosities low enough for drop-based bioprinting (Blaeser et al., 2017) and have been proven to be stable tumor model materials (Lake et al., 2011; Lee and Cho, 2016; Campos et al., 2019b). Agarose was chosen for its simple handling, low cost, superior printability and print resolution, though it has failed in certain tissue models such as muscle and vasculature due to its lack of cell adhesion motifs (Köpf et al., 2016). Collagen I is a popular material in tissue models, as it is the most abundant ECM component in the human body and positively influences cell morphology and proliferation (Patterson et al., 2010), but its slow gelation kinetics prevent precise and complex printing (Parenteau-Bareil et al., 2010; Patterson et al., 2010; Duarte Campos et al., 2016; Jia et al., 2016). As a third material, gelatin was selected as an additive for agarose and collagen. While it is not stable under cell culture conditions in its native form, its amphiphilic composition and use as foam stabilizer can support drop formation, gelation kinetics and adjust the rheological properties of the bioink (Erkoc et al., 2020; Piluso et al., 2021).

Cell interaction and post-printing cell behavior were studied for a liver carcinoma model based on HepG2 cells. HepG2 is a hepatoblastoma cell line popular for bioprinted liver models and as cancer drug models (Chang et al., 2010; Bhise et al., 2016; Lee and Cho, 2016; Lee et al., 2017; Zhu et al., 2017; Ma et al., 2018; Fritschen and Blaeser, 2020; Maloney et al., 2020; Sun et al., 2020). They are robust cell line, greatly available and simple to culture (Müsch et al., 2020; Štampar et al., 2020; Kammerer, 2021; Lv et al., 2022), making them the most widely used hepatoma cell line (Donato et al., 2013; Arzumanian et al., 2021). Additionally, they exhibit a tumor spheroid-like structure when cultured in 3D and good colony-forming potential (Luckert et al., 2017; Chen et al., 2021). In summary, we developed a thorough characterization methodology to evaluate different bioinks for their suitability for 3D-bioprinted liver cancer models (Figure 1). We could determine the porosity, stiffness, diffusivity, viscosity, drop shape, size and wetting for seven blends of agarose, collagen and gelatin. Cell-material interaction and post-printing cell behavior were studied for a liver carcinoma model based on HepG2 cells. This work presents a summary of the characterization procedure and the properties that need to be taken into consideration when choosing the optimal hydrogel for microvalve-based Drop-on-Demand (DoD) bioprinting.

## 2 Results and discussion

### 2.1 Gel structure

**FIGURE 2:**
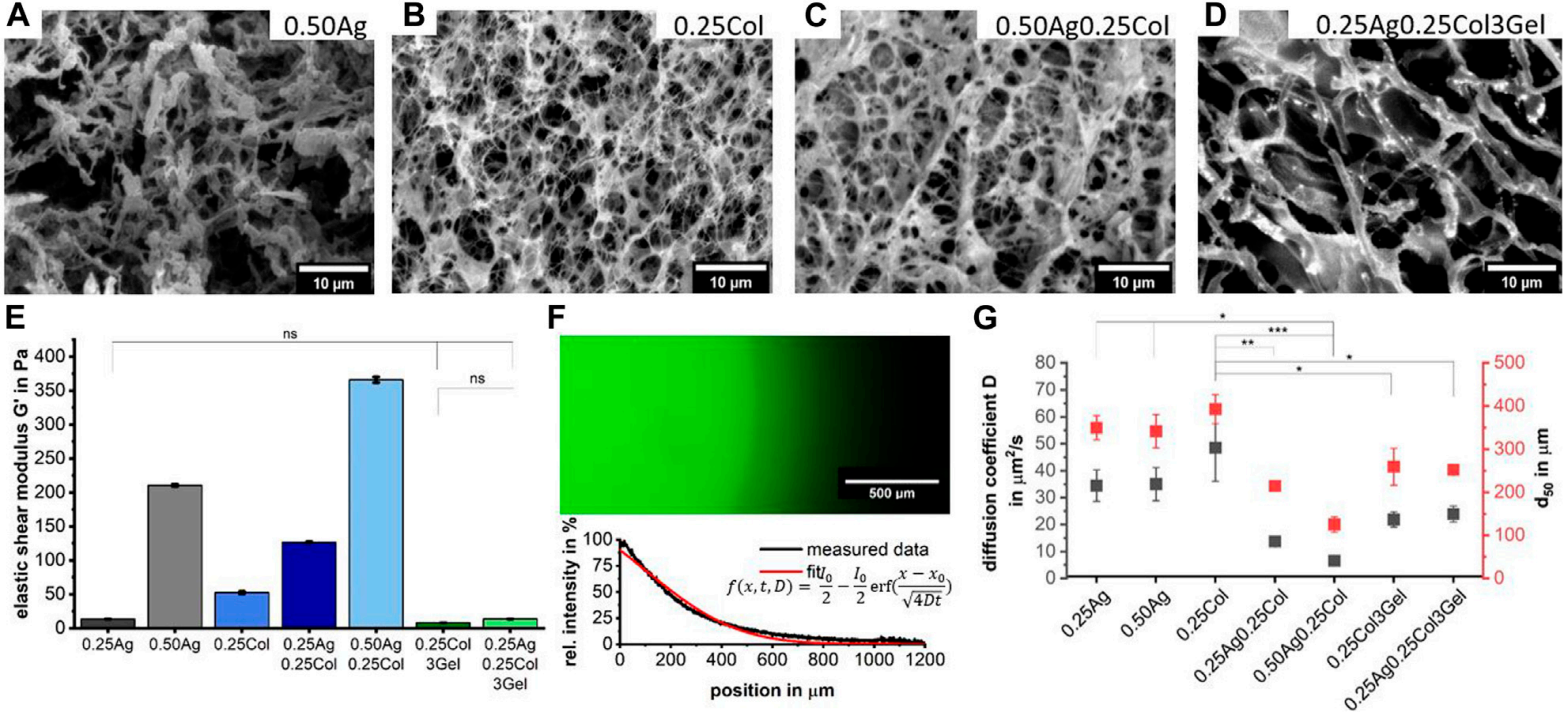
SEM images show the microstructure of 0.50Ag **(A)**, 0.25Col **(B)**, 0.50Ag0.25Col **(C)** and 0.25Ag0.25Col3Gel **(D)**. The elastic component of the shear modulus G′ for all seven materials as measured in a rotational rheometer; *p* < 0.001 except when stated otherwise; n = 3 **(E)**. The diffusion of FITC-labelled bovine serum albumin was determined using Fick’s second law of diffusion on fluorescence intensity profiles after 30 min of incubation as shown for 0.50Ag **(F)**. From these profiles, the diffusion coefficient D and the distance until 50% signal intensity remained $d_{50}$ were determined; *p* < 0.001 for D unless stated otherwise; $d_{50}$ was found unsignificant; n = 3 **(G)**.

The microstructure as well as the mechanical properties of hydrogel blends comprising agarose, collagen and gelatin (see Table 3 for abbreviations and nomenclature) were determined (Figure 2). SEM-images reveal that the collagen fibers are homogeneously distributed in the agarose matrix. SEM-images indicate a decreasing pore size with increasing total polymer concentrations for agarose-collagen blends (Figure 2C), in accordance with previous reports (Ulrich et al., 2010; Köpf et al., 2016; Quarta et al., 2021). In contrast, the addition of gelatin leads to larger pores, which is especially dominant for 0.25Ag0.25Col3Gel (Figure 2D). This effect can be attributed to the post-fabrication liquefication of gelatin domains at 37°C, leaving gaps in the agarose-collagen network.

The mechanical properties of hydrogels are greatly determined by their crosslinking density, monomer backbone and porosity (Anseth et al., 1996; Normand et al., 2000; Yang and Kaufman, 2009; Quarta et al., 2021). In particular the latter is known to influence cell morphology, proliferation, migration and differentiation (Engler et al., 2006; Skardal et al., 2013). In addition, the material’s elastic behavior is also important in bioprinting, as a high elastic modulus offers good shape fidelity and overall stability over longer cultivation times (Parak et al., 2019). Experimentally, the average elastic component of shear modulus G′ was determined around the linear viscoelastic region of the strain curve with a rotational rheometer. As expected (Duarte Campos et al., 2019), higher concentrated hydrogels such as 0.50Ag and 0.50Ag0.25Col offer the highest shear modulus of 211 and 366 Pa, respectively (Figure 2E). Interestingly, the blend of agarose and collagen leads to shear moduli that are higher than the sum of the individual components (Figure 2E). This effect might be attributed to an increased collagen fiber diameter in collagen blends as observed in the according SEM-images (Figures 2A, D; Supplementary Figure S1), which has been reported before for collagen polymerized at lower temperatures (Yang and Kaufman, 2009; Achilli and Mantovani, 2010; Holder et al., 2018; Cambria et al., 2020). These conditions apply in agarose-collagen blends, since neutralizing of gels occurs at 20°C compared to 37°C for only collagen containing gels (see Material and Methods). Blends containing gelatin possess a much lower shear modulus, which can be explained by the previously described pore formation by gelatin liquefication (Figure 2E).

The microstructure of gels also impacts the oxygen and nutrient diffusion as well as waste removal (Breslin and O’Driscoll, 2013). The diffusion of FITC-labelled bovine serum albumin (BSA), the most abundant component of serum-containing media and critical in nutrient transport (Francis, 2010), was measured with fluorescence intensity profiles that were taken after 30 min of incubation in cell-free hydrogels. Fick’s second Law of Diffusion was fitted to the measured curves and the diffusion coefficient D and the distance of 50% signal intensity $d_{50}$ were obtained (Figures 2F,G). Pure agarose and collagen showed the best diffusivity and a diffusion distance of 350–400 μm, while blends of agarose and collagen exhibit only half of these values, which correlates to the denser fiber networks and increased shear modulus observed previously. As discussed before, the addition of gelatin leads to formation of bigger pores, explaining an increased BSA diffusion, although still lower than for native agarose or collagen.

Of all seven materials, 0.50Ag shows the most promising microstructure and mechanical properties. It has larger pores and a BSA diffusion-limit of 300 µm comparable to native tissue (Macdougall and Mccabe, 1967; Jain et al., 2005). Its comparably higher shear modulus should facilitate 3D-bioprinting of complex but stable tissue models.

### 2.2 Rheological properties and gelation

**FIGURE 3:**
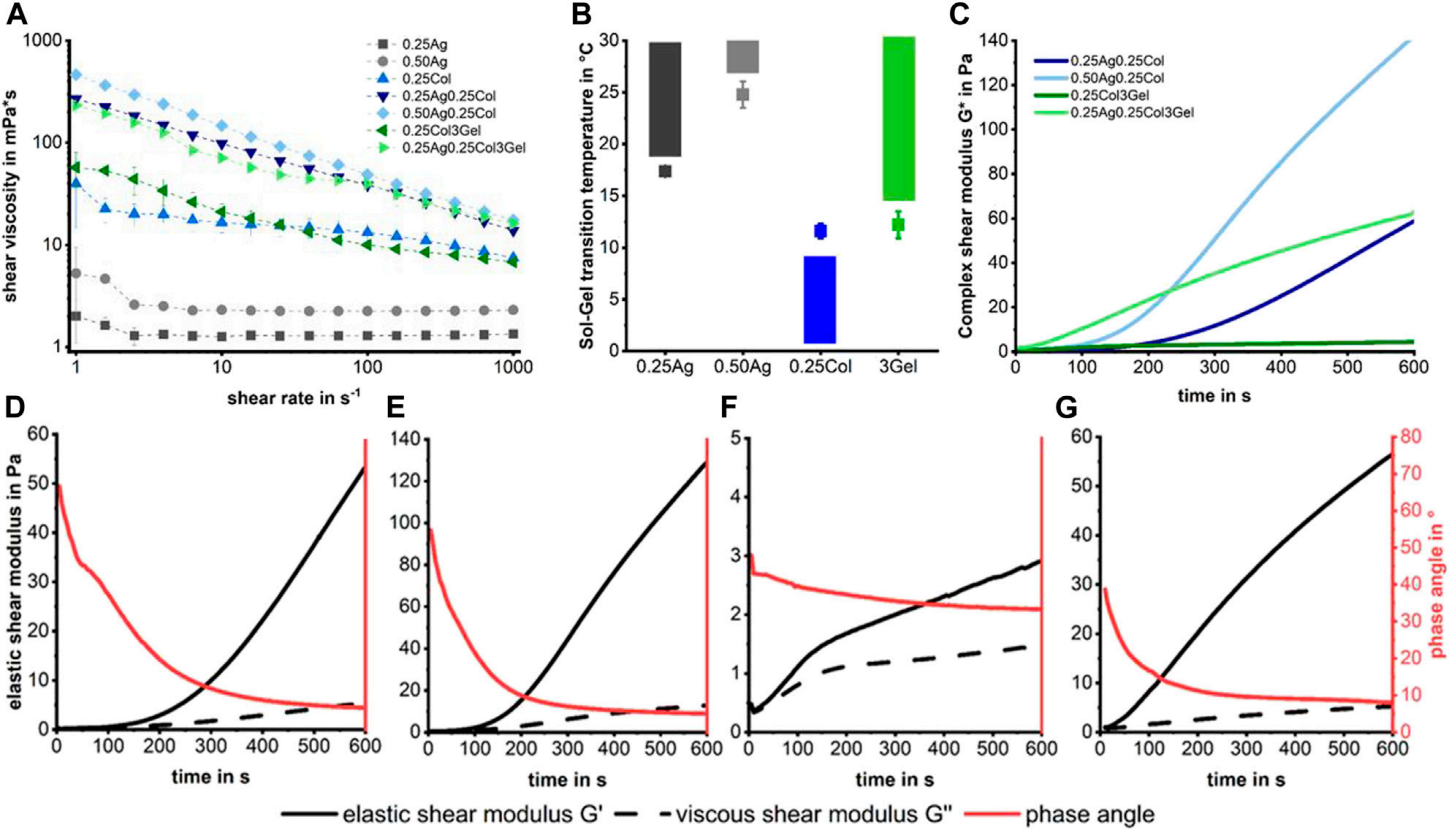
Shear viscosity measurements show Newton-like behavior for agarose gels, while collagen containing gels are strongly shear thinning **(A)**. No fabrication window is visible in the sol-gel transition temperatures **(B)**, meaning that collagen starts to polymerize in a liquid matrix of agarose or gelatin, leading to an increasing shear modulus over time **(C)**. The gelation kinetics for each mixture of agarose/gelatin and collagen show an increasing modulus and reduced phase angle over time for 0.25Ag0.25Col at 20°C **(D)**, 0.50Ag0.25Col at 25°C **(E)**, 0.25Col3Gel at 20°C **(F)** and 0.25Ag0.25Col3Gel at 20°C **(G)**. n = 3 for **(A,B)** with standard deviation.

While of little importance for cell culture, the rheological properties of the presented materials are crucial for handling and use in 3D-bioprinting (Blaeser et al., 2016). A higher shear viscosity implies smaller, well-shaped drops with high post-printing shape fidelity, while inflicting a higher shear stress for cells during the dispensing process (Blaeser et al., 2017) and increasing the risk of nozzle clogging. Shear thinning properties are favorable as the decrease in shear viscosity during the printing process may increase post printing cell viability. Both 0.25Ag and 0.50Ag exhibit low shear viscosities of 1.3 and 2.3 mPa*s respectively with Newtonian like flow behavior (Figure 3A). In contrast, all blends containing collagen have higher shear viscosities of up to 450 mPa*s at low shear rates and a shear thinning behavior, caused by collagen fibers aligning under shear. This shear thinning effect is enhanced when agarose or gelatin are added, probably caused by the onsetting gelation of collagen fibers, as has been reported before (Köpf et al., 2016; Stratesteffen et al., 2017). While agarose-collagen blends exhibit rather high shear viscosities at low shear rates, their high shear thinning properties imply that their viscosity will be in a similar range to agarose at shear rates found inside the nozzle of a bioprinter, which ranges at around 500,000 s^-1^.

The sol-gel transition temperature of the primary hydrogels determines the fabrication windows under which each material can be processed (Figure 3B). The results show that there is no fabrication window at which both collagen and agarose or gelatin are liquid over a longer period, as collagen starts to gel at temperatures above 10°C, which is below the transition temperature for the polysaccharide. For combinations containing collagen, we therefore decided to handle the blends at temperatures in the sol-state of agarose or gelatin as these materials gel rapidly below their gel-temperature. The fiber formation and gelation of collagen does progress at 20 or 25°C, leading to an increasing shear modulus over time (Figure 3C) and providing a short time of handling. The formation of collagen fibers starts within seconds after neutralization, reducing the phase angle measured in the rheometer below 45° within 1–4 min at a steadily increasing shear modulus. This effect is slowest for 0.25Col3Gel and fastest for 0.25Ag0.25Col3Gel (Figures 3D–G). These blends therefore require a highly controlled temperature handling and very fast processing to prevent collagen fibers to fully polymerize or the gel to solidify.

In conclusion, it can be stated that no stable process window can be found due to the contrasting gelling behavior of the individual blend components. However, their different gelling kinetics open up a defined time window in which processing by means of 3D bioprinting is nevertheless possible. Depending on the process temperature and the polymer content, this window lies between 3 and 10 min. The challenges in handling require skilled workers, precisely planned experiments and allow only for limited printing times.

### 2.3 Printability

**FIGURE 4:**
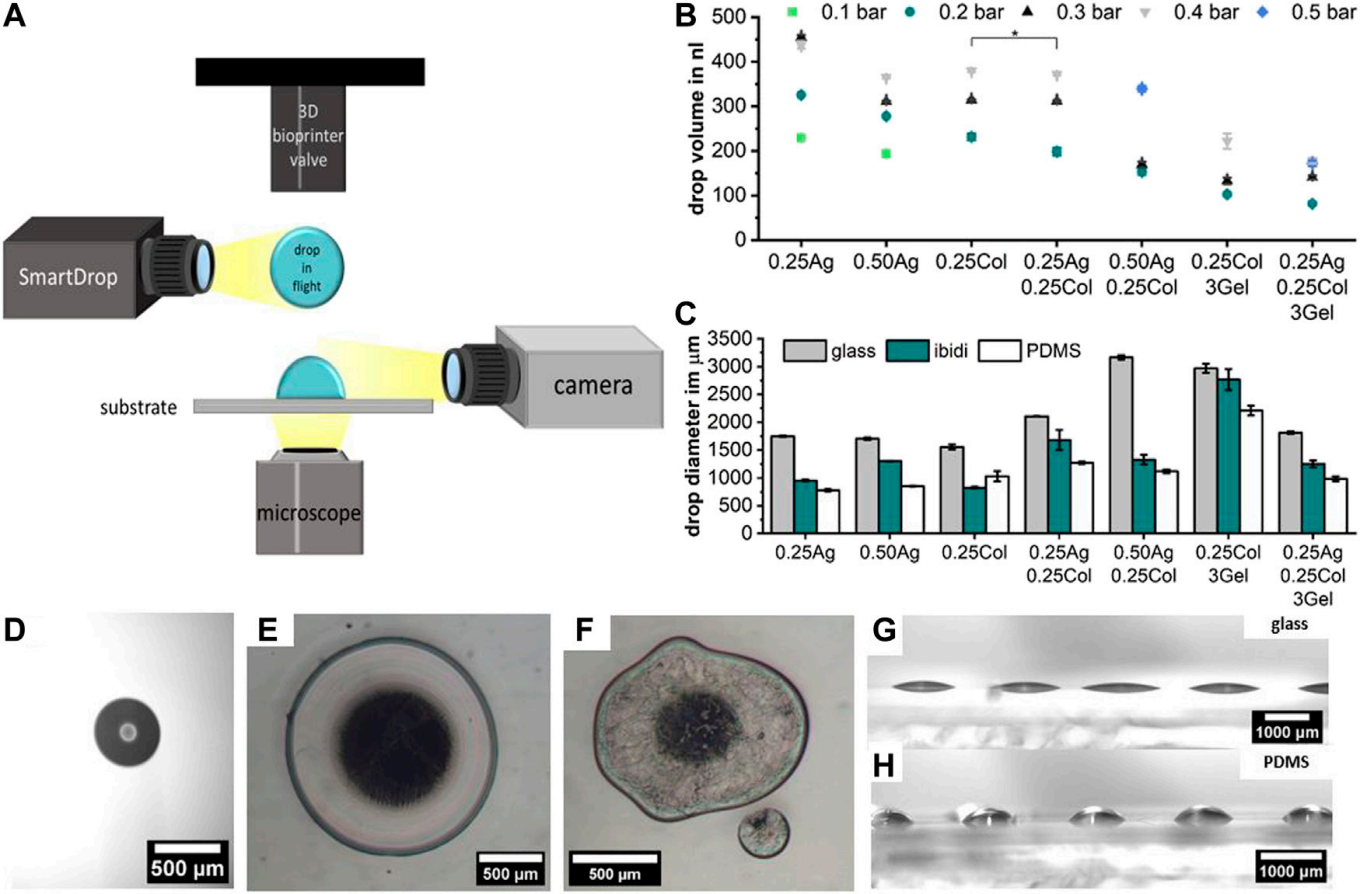
Schematic representation of the optical characterization of hydrogel blend printability, including the drop volume in flight using an embedded “SmartDrop” system, the effective drop diameter after printing on various substrates using an optical microscope, and the wetting behavior observed by camera **(A)**. The drop volume for different print pressures measured using the “SmartDrop” system; with *p* < 0.001 unless stated otherwise, number of samples for each data point is in Supplementary Figure S2 **(B)**. The drop diameter on three different substrates measured using an optical microscope is given for the minimum printable pressure in bar noted on top of the bars **(C)**. Example image of the drop volume detection in flight **(D)**. Microscopy of drops of 0.25Ag **(E)** and of 0.25Ag0.25Col3Gel **(F)** printed on PDMS at 0.2 bar clearly show the difference in roundness and the occurrence of satellite drops for AgCol-blends. The difference in effective drop area for different substrates is caused by the wetting behavior, as shown for 0.50Ag on glass **(G)** and PDMS **(H)** printed with 0.2 bar print pressure; n = 10.

Studying the printability of hydrogels for Drop-on-Demand (DoD) printers is crucial for the fabrication of high-resolution tissue models. While the rheological measurements provide an objective view on the materials’ flow behavior, the printability study takes additional effects such as the droplet formation during dispensing and the wetting behavior at the drop substrate interface into account. The drop formation and wetting behavior were measured with a tri-optical approach: drop volume in flight using an embedded “SmartDrop” system, the effective post-printing drop diameter on a substrate measured by microscopy, and the wetting behavior on different substrates observed by camera (Figure 4A, Supplementary Figure S2 Video). All prints were conducted on a microvalve-based DoD bioprinter, which has been described in detail before (Blaeser et al., 2016), with applied print pressures given in Table 2.

In flight, the drop volume increases with increasing print pressure as expected. The lowest print pressure without nozzle clogging depends on the material viscosity, but results in a drop volume between 100–200 nl for all seven materials (Figures 4B, D, Supplementary Figure S2). As expected from the rheological measurements, not all materials were easily printable. When printing agarose-collagen blends (0.25Ag0.25Col and 0.50Ag0.25Col) splashing and irregular drop shapes post-printing often became visible under the microscope. Drops of pure agarose and collagen exhibited round and regular shapes (Figures 4E, F). Pure materials are therefore preferable over blends if an easy and clean print results is desired, as the onsetting formation of collagen fibers during handling in blends most likely reduces the print quality and becomes problematic in long-time printing of more complex geometries.

The lateral print resolution, determined by the effective drop diameter after printing, is dominated not only by the hydrogel’s viscosity, but also by the substrate’s surface energy (Figure 4C). A drop of a certain volume resulted in a large, flat drop with a low effective contact angle on glass (Figure 4G). A drop of the same material and volume had a smaller diameter and more spherical shape on poorly wettable surfaces such as PDMS (Figure 4H), as observed by camera. For highly resolved printing, a substrate with a high contact angle is preferrable, though the hydrogel-hydrogel wettability becomes dominant in multi-layered prints.

All presented materials are generally suitable for microvalve based bioprinting. In order to select the blend of choice, the geometry of the final tissue model should be taken into consideration. While 0.25Col exhibited a better printability and rounder drop shape, its long gelation time of over 5 min at 37°C limits its application for multi-layered structures or multi-material printing. In contrast, agarose gels rapidly at room temperature, so larger and complex print geometries can be printed at high print quality and low fabrication times. When collagen is blended with agarose or gelatin, the rapid gelation holds true, which is a key advantage of printing with blends compared to pure collagen. However, the onsetting fibrillogenesis of collagen in blends greatly limits the time for printing to a few minutes, with less reproducibility and a risk of nozzle clogging. This can be countered by increasing the print pressure to prolong print times at the cost of print resolution.

### 2.4 Cellular behavior in gels

**FIGURE 5:**
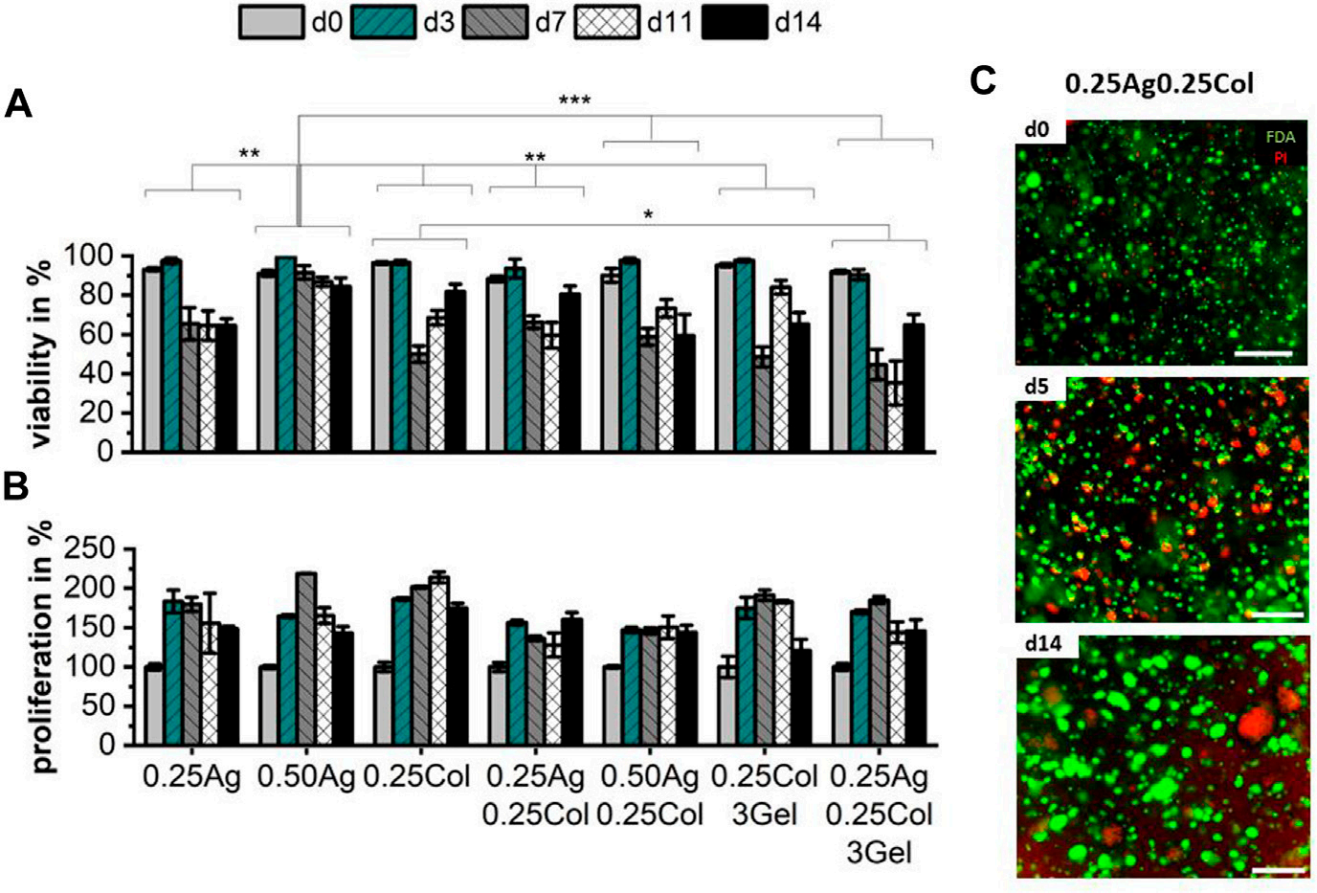
Cell viability **(A)** and proliferation **(B)** of 1 × 10^6^ cells/ml of HepG2 cells in agarose, collagen and gelatin blends over the course of 14 days; n = 3 with ns for no significance, * for *p* < 0.05, ** for *p* < 0.01 and *** for *p* < 0.001. Exemplary images of HepG2 in 0.25Ag0.25Col stained with FDA for live (green) and PI for dead (red) cells on days 0, 5 and 14 show increasing size and number of agglomerates with necrotic cores **(C)**. Scale bar showing 500 µm.

It is well known that among other hydrogel properties, the stiffness, porosity, pH-level, chemical composition and the presence of cell adhesion ligands impact cell morphology and behavior (Breslin and O’Driscoll, 2013). The selected hepatoblastoma cell line HepG2 exhibits a high (>80%) cell viability during the first 3 days of culture in all seven materials. Viability was maintained in 0.50Ag only, dropping below 60% for all other blends after 14 days in static culture, with great variance inside measured samples (Figure 5A). This drop in cell viability may be caused by an increasing agglomeration of cells over time in the gel, as can be expected from a cancer cell line (Luckert et al., 2017; Kammerer, 2021). Over time, cells formed agglomerates with a necrotic core in the center as visible in the live/dead staining images as shown for 0.25Ag0.25Col (Figure 5C). The formation of cell agglomerates may be the cause for the reduced measured cell viability, while a higher number of live cells are found on the outer layers and in smaller clusters. Further testing still needs to be done to confirm this hypothesis.

At the same time, proliferation increases during the first days to up to 200%, but decreases or stagnates afterwards (Figure 5B), with no significant difference between materials. The rather low metabolic activity compared to 2D culture has been reported before in literature (Štampar et al., 2020). This effect is most likely caused by a reduced cell division rate while the cells continue to arrange in 3D and form the mentioned agglomerates, with viable and active cells on the outside and an inner necrotic core. In contrast to other cell types and cell lines, which prefer hydrogels of a certain chemical composition, porosity or elasticity, no distinct difference in viability or proliferation of HepG2 in the studies materials can be observed. The latter might be explained by the carcinomic characteristics of HepG2.

**FIGURE 6:**
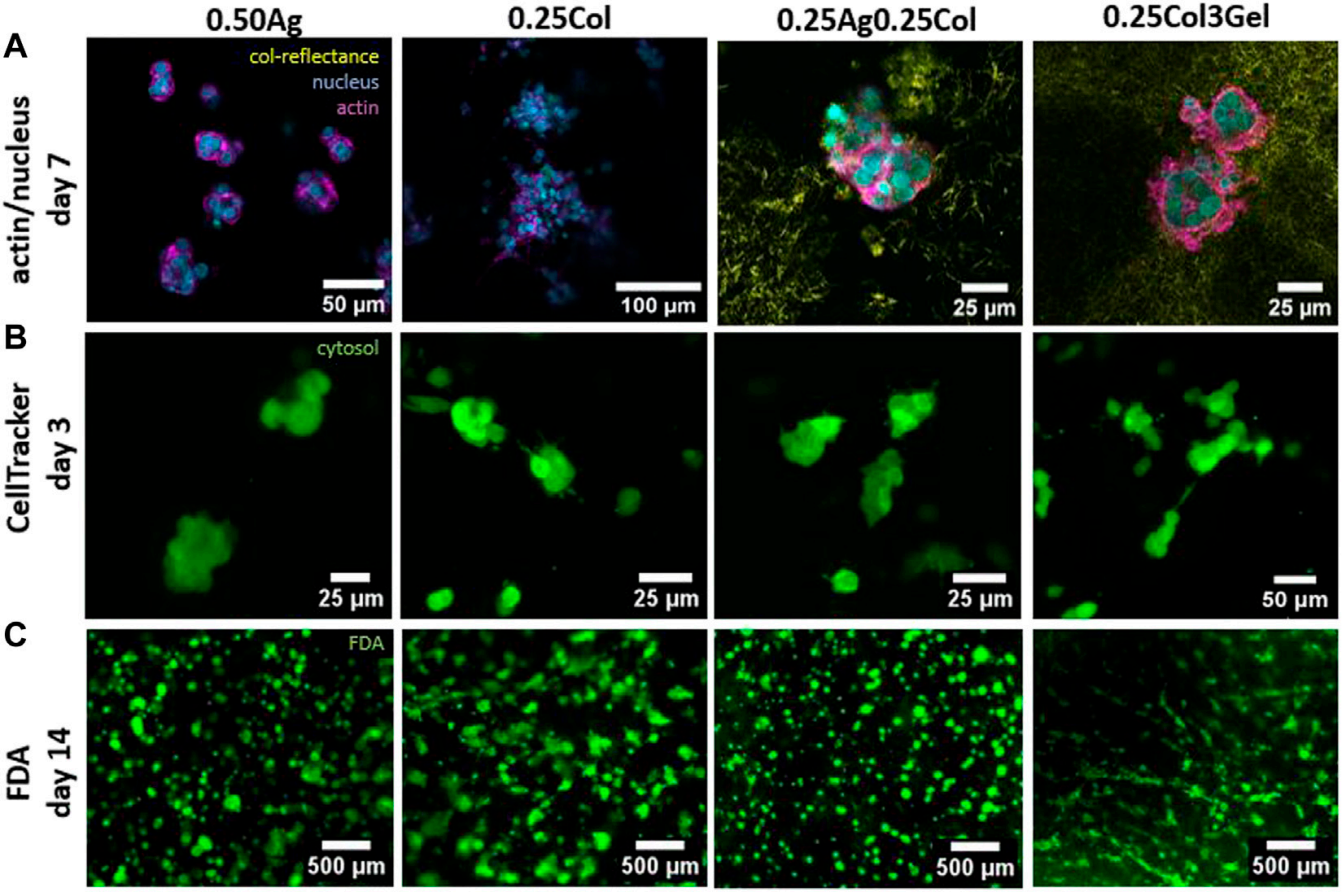
Confocal fluorescence microscopy images of HepG2 cells in 0.50Ag, 0.25Col, 0.25Ag0.25Col, 0.25Col3Gel with actin filaments (magenta) and cell nuclei (blue) stained on day 7 and collagen fibers visualized by confocal reflectance (yellow) **(A)**. Images of HepG2 cells marked green with CellTracker on day 3 **(B)** and live cells stained green with FDA on day 14 **(C)**.

Small morphological differences arise during culture. HepG2 have very rounded shape with a dominant amount of cell mass given by nucleus and little actin or cytosol (Figure 6A), with cells arranged as agglomerates of 4 cells and more. Since 0.25Ag and 0.50Ag offer no cell attachment sites, the cells exhibit spherical shapes. Blends that contain collagen show small protrusions of actin and cytosol visible when stained for actin or labelled with CellTracker green (Figures 6A, B; Supplementary Figure S4), which has been reported in carcinoma cells before (Campos et al., 2019b). The length of these protrusions increases in 0.25Col and 0.25Col3Gel after 14 days of culture, where cells start to build up a small network structure (Figure 6C). Both 0.25Col and 0.25Col3Gel offer the lowest elastic modulus and a highly fibrous networks, which could explain the difference in cell morphology.

During the first days in culture, HepG2 exhibit a similar shape and size in all materials, unlike most other cell types which exhibit a strong contrast when cultured in agarose compared to collagen containing gels (Ulrich et al., 2010; Köpf et al., 2016; Campos et al., 2019b). This could be caused by a low affinity to remodel the surrounding matrix, as confocal reflectance images visualizing the distribution of the cells in and around collagen fibers indicate (Figure 6A). Consistent with previous reports (Nguyen et al., 2022), collagen fibers show a homogeneous distribution, both inside agarose and gelatin matrices, with little remodeling around the cells after 7 days in culture. A change in cell morphology was however observed for blends of collagen and gelatin, where cell spreading occurred after 10 days in culture. The otherwise lacking matrix remodeling and morphological differences could explain the small effect of the material choice on HepG2 viability and proliferation.

### 2.5 Bioprinting of HepG2 cells

**FIGURE 7:**
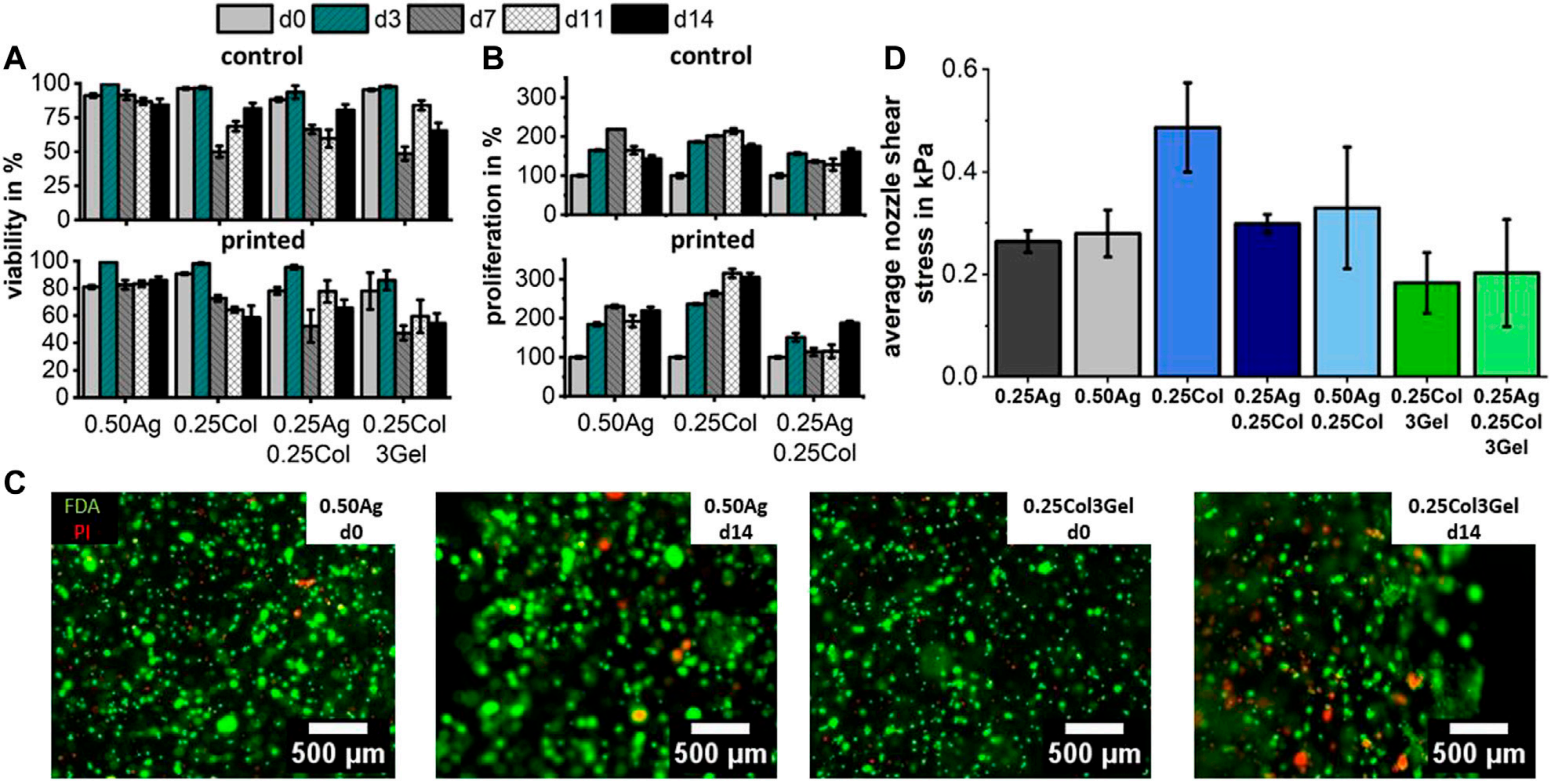
Cell viability **(A)** and proliferation **(B)** for 1 × 10^6^ cells/ml HepG2 cells in agarose, collagen and gelatin blends both after printing and for a control group. Fluorescence images of bioprinted HepG2 stained with FDA for live (green) and PI for dead (red) cells show no negative effect of printing on cell viability after 14 days in culture **(C)**, which corresponds to the low nozzle shear stress that cells encounter during printing **(D)**. Cell viability and proliferation were found unsignificant between printed and control group, except for viability in 0.25Col3Gel (*p* < 0.01); n = 3.

Following the described blend characterization and printability evaluation, the influence of the printing process on HepG2 cells was studied. The post-printing response of HepG2 cells is exemplarily shown for four materials that offered the best results in the previous experiments and which are promising candidates for microvalve-based bioprinted liver cancer models. In comparison to the non-printed control (NP) group, cell viability and proliferation were not affected significantly by the print process, except for viability in 0.25Col3Gel (*p* < 0.01) (Figures 7A–C). The same observations were made for the three remaining materials as illustrated in the (Supplementary Figure S5).

The observed high post-printing viability matches the expected values based on the calculated average shear stress $\bar{\tau }$ inside the printer’s nozzle that cells experience during printing. The estimated average nozzle shear stress was shown to be in a range from 0.2–0.3 kPa for all materials, except for 0.25Col with approx. 0.5 kPa (Figure 7D). These values are far below reported critical shear stresses, which lie in ranges above 2–5 kPa (Blaeser et al., 2016).

In conclusion, all seven blends can generally be considered viable candidates for microvalve-based DoD bioprinting of HepG2 cells with high post-printing viability and proliferation potential. This provides future users with a wide range of printable materials to choose from. By adjusting the polysaccharide and protein concentration, the mechanical properties and diffusion behavior of the material as well as the precision of the printing process can be precisely adjusted. However, it should be noted that the bio-chemical equality of material selection - only small morphological differences with no significant impact on viability and proliferation - solely applies to the tumor cell line (HepG2) investigated in this study. It is to be expected that other cell types, especially primary cells, react significantly more sensitively to a change in the physicochemical milieu.

## 3 Summary and outlook

A methodology to study the differences in material properties of agarose, collagen and gelatin blends with a focus on microvalve-based DoD bioprinted, 3D tissue models was developed. The three hydrogels were selected for their availability, printability and cell activity and were thoroughly characterized. The microstructure was shown to influence the stiffness of the gels. Moderately concentrated agarose and blends thereof with collagen exhibited the highest elasticity. Gels of higher polymer concentration exhibited a denser and less porous microstructure, and thus, a lower diffusivity for small molecules such as albumin. However, blends that comprised low agarose concentrations (e.g., 0.25Ag and 0.25Ag0.25Col3Gel) did not remain stable during cell culture, making them unsuitable for bioprinting applications, which require long-term culture conditions.

Rheological measurements show great differences in shear viscosities, but also in shear thinning behavior and in gelation. It was shown that there is no fabrication temperature in which both collagen and agarose or gelatin are in a stable solution state. Since collagen gelation sets on within seconds, the handling times of bioinks before and during printing was limited to 5–10 min. Despite their different rheological behavior, all of the selected bioinks could be printing with a drop-on-demand microvalve 3D-bioprinting system. Using a newly developed “SmartDrop” system, the volume of single drops in flight could be measured. The smallest drops achieved had a volume of 100–200 nl at lower print pressures. Greater differences between blends were visible once the resulting drops were imaged under the microscope. Pure agarose and collagen gels formed round drops, while drops of blends exhibited satellite drops, splashing and irregular shapes. The difference is most likely caused by the onsetting gelation of collagen fibrils in blends, which make handling and printing more complex. It is therefore expected of the blends to offer a reduced effective print resolution due to their shape irregularity.

Testing these materials on a tissue model of HepG2 cells, less distinct differences between the presented materials became present in contrast to other cell types. No significant difference in viability or proliferation was observed. However, cells developed small protrusions and actin fibers only when cultured in collagen-containing gels. At the same time, cells retained a very round shape dominated by little cytosol, formed agglomerates and did not remodel their surroundings over the course of 14 days in all materials. Greater differences in cell-biomaterial interaction are expected for other cell types, especially for primary sourced cells. Combining the previous results, 3D-bioprinting experiments showed no influence on cell viability or proliferation for all materials, which corresponds to the very low shear stress below 0.4 kPa that cells experience during printing. Because of the low shear stress, the same high viability is expected for other cell types as well, including primary and stem cells (Blaeser et al., 2016). A summary of all results obtained in this study is given in Table 1. The rating is based on the materials used in this study and compares the strengths and weaknesses of each.

**TABLE 1:** Summary of material properties as tested in this work. Qualitative ratings as a comparison between materials are given with ++ (very good), + (good), o (neutral), - (poor) and - - (very poor).

	0.25Ag	0.50Ag	0.25Col	0.25Ag 0.25Col	0.50Ag 0.25Col	0.25Col 3Gel	0.25Ag 0.25Col 3Gel
Handling	+ +	+ +	+	-	-	o	- -
Printability		+ +	+	-	-	o	-
Stability during culture	-	+	+	++	++	+	-
Nozzle shear stress during printing	++	++	++	++	++	++	++
Shape fidelity/gelation	o	+	- -	+	+ +	-	+ +
Diffusivity	+	+	+ +	-	- -	o	o
Biofunctionality: cell adhesion sites Das and Basu (2019); Parak et al. (2019); Unagolla and Jayasuriya (2019)	- -	- -	+ +	+	+	+	+

With the profound characterization of the seven materials, the gained knowledge can be applied onto 3D-bioprinted liver cancer models. With a specific application and its requirements regarding cell-material interaction and print complexity in mind, a thought-out selection of one of the presented materials with its strengths and weaknesses is easy. For applications requiring complex geometries with a focus on bioprinting, 0.50Ag has proven high printability, stability and easy handling. When cell migration, invasion or vascularization is a key component, 0.25Col and 0.25Col3Gel are recommended. If shape fidelity or printing speed is required, blending agarose and collagen can combine the advantages of both, whilst adding a certain level of complexity in handling.

The presented results can also be translated to other cell types and tissue models. Only the study of cell-material interaction would be required for non-cancerous cell types such as primary hepatocytes, myoblasts or fibroblasts. The work described presents a robust methodology to characterize biomaterials for bioprinting. As the choice of bioink component is critical to the success of a bioprinted tissue model, standardized characterization and evaluation procedures ensure the optimum is chosen. This leads the way to a faster and more effective development of bioprinted tissue models.

Interestingly, no significant cell biological differences were found between the materials studied with respect to morphology, viability and proliferation potential. However, this observation is only due to the tumor cell line used (HepG2). For similar cell types, such as epithelial-like carcinoma or hepatic-like cancer cells, even rather inert hydrogels that lack the presence of cell adhesion ligands could potentially be a promising choice. This includes native agarose with its ease of use, its excellent drop forming potential and the resulting high printing precision. However, the extent to which this matrix environment, which differs in physico-chemical structure from native ECM, influences tumor tissue formation and accompanying pathological cell-cell signaling pathways remains to be determined in future studies.

## 4 Materials and methods

### 4.1 Hydrogel composition

To prepare the stock solutions, agarose (low gelling temperature, Sigma-Aldrich, St. Louis, United States of America) was mixed with distilled water and sterilized in the autoclave. Before use it was reheated to 70°C and maintained at 40°C. Gelatin (IMAGEL LA, Gelita AG, Eberbach, Germany) was mixed with distilled water in a 40°C water bath and sterile filtered using a cellulose acetate 0.2 µm syringe filter (VWR, Radnor, United States of America). Gelatin stock was warmed to 37°C before use. Sterile bovine collagen type I (FibriCol, Advanced Biomatrix, Carlsbad, United States of America) was stored cold until use.

Seven hydrogel blends were prepared directly before use by mixing stock solutions of agarose, gelatin and bovine collagen I in the order listed in Table 3. The gels containing collagen were neutralized with 1M NaOH solution (Sigma-Aldrich, St. Louis, United States of America). For experiments containing HepG2 cells, 175 µl of DMEM were replaced by a HepG2 stock suspension with a final concentration of 1 × 10^6^ cells/ml.

### 4.2 Microscopy

Bright field, phase contrast and fluorescence microscopy were conducted with a light microscope (Echo Revolve, Discover Echo Inc. San Diego, United States of America). Confocal laser scanning fluorescence microscopy images were taken on a TCS SP8 microscope (Leica Microsystems, Mannheim, Germany). Prior to SEM imaging, samples were freeze-dried overnight and sputtered with a 15 nm layer of Pt/Pd. Images were acquired at 4 kV and at 400, 1500, 5000 and 10000 times magnification with the Zeiss Evo 10 (Carl Zeiss AG, Oberkochen, Germany).

**TABLE 2:** Print pressure and valve opening time for 3D-bioprinting experiments conducted with all materials, with and without cells.

**Material**	Cell printing	Drop volume	Drop area
	**Print pressure in bar**		
0.25Ag	0.3	0.1–0.4	0.1–0.4
0.50Ag	0.3	0.1–0.4	0.1–0.4
0.25Col	0.4	0.2–0.4	0.3–0.5
0.25Ag0.25Col	0.3	0.2–0.4	0.2–0.5
0.50Ag0.25Col	0.5	0.2, 0.3, 0.5	0.2–0.5
0.25Col3Gel	0.3	0.2–0.4	0.2–0.5
0.25Ag0.25Col3Gel	0.5	0.3–0.6	0.3–0.6

### 4.3 Diffusion of FITC-albumin

Diffusion of nutrients was simulated with FITC-labelled BSA (Sigma-Aldrich, St. Louis, United States of America) at a concentration of 50 μg/ml in PBS. 30 µl of each material were added through one side of an Ibidi 0.4µ-Slide (ibidi GmbH, Gräfelfing, Germany) and allowed to gel (n = 3). FITC-BSA (50 µl) was added through the other side and incubated for 30 min at room temperature. Fluorescence imaging was done with a light microscope. Intensity profile lines were taken at each time point using ImageJ starting at the gel-liquid interface. The diffusion coefficient was determined by fitting the fluorescence intensity profile using Fick’s second Law of Diffusion. The diffusion distance d_50_ of FITC-BSA was set at the point where only 50% of the initial signal intensity was reached.

### 4.4 Rheological characterization

Rheological measurements were conducted on a rotary oscillating rheometer (Kinexus lab+, NETZSCH-Gerätebau, Selb, Germany). The shear viscosity was measured using a 1° cone-plate geometry with diameter of 60 mm for shear rates from 1 to 1000 s^-1^, taking five measurement points per decade. Agarose gels were measured at 37°C, collagen at 10°C and mixtures of collage with agarose or gelation were measured at 20°C, except for 0.50Ag0.25Col, which was measured at 25°C. For each hydrogel formulation, three shear rate curves were taken.

Sol-Gel transitions were measured at a temperature rate of 1°C/min three times per material at a frequency of 1 Hz using a plate geometry with a diameter of 40 mm with a 0.5 mm gap. Gelation kinetics were measured at 20°C for mixtures of collagen with agarose or gelatin, except for 0.50Ag0.25Col which was measured at 25°C. All measurements were conducted with a constant shear strain of 2% at a frequency of 1 Hz for 10 min.

For shear moduli measurements, samples containing agarose or gelatin were allowed to gel at 10°C for 10 min. For mixtures with collagen, the temperature was also increased to 37°C for another 30 min. Shear strains from 0.01% to 100% were sampled with a frequency of 1 Hz at 10 sample points per decade. Measurements were repeated three times for each hydrogel formulation.

### 4.5 Cell culture

Human liver carcinoma cells (HepG2, ATCC, Manassas, United States of America) were cultured in low glucose DMEM (Gibco, Life Technologies Limited, Parsley, United Kingdom) with 10% FBS (Thermo Fisher Scientific Inc. Waltham, United States of America), 1% PenStrep and 1% Amphotericin B (Gibco, Life Technologies Limited, Parsley, United Kingdom), needing passage every third or fourth day.

### 4.6 Cell assays

For all cell assays, HepG2 at passages below 10 were seeded in the gel at a final concentration of 1 × 10^6^ cells/ml and experiments carried out for n = 3. Proliferation of HepG2 in culture was assessed with a CellTiter Blue assay (Promega Corporation, Fitchburg, United States of America). 100 µl of medium with 20 µl CellTiter Blue were added to each well and incubated at 37°C for 3 h. Fluorescence intensity of the supernatant was read with an Infinite M Plex plate reader (Tecan Group AG, Männedorf, Switzerland). Cell viability was determined by staining for live cells with fluorescein diacetate (Sigma-Aldrich, St. Louis, United States of America) and dead cells with propidium iodide (Carl Roth GmbH + Co. KG, Karlsruhe, Germany) (1:60 diluted in Ringer’s solution). Quantification of viable and dead cells was done in ImageJ (see Supplementary Figure S3).

For the analysis of live cell morphology inside the gels, HepG2 were incubated with 2 µM CellTracker™ Green CMFDA Dye (Thermo Fisher Scientific Inc. Waltham, United States of America) per 10 × 10^6^ cells for 30 min before seeding. Cells retained their fluorescence signal and passed it on to daughter cells for up to 7 days. Before immunofluorescence stains, cells were fixed in 4% paraformaldehyde (Carl Roth GmbH + Co. KG, Karlsruhe, Germany) for 15 min and permeabilized with 0.5% Triton X-100 in PBS (Carl Roth GmbH + Co. KG, Karlsruhe, Germany) for 10 min. Actin filaments were stained for 30 min with Alexa Fluor 488 Phalloidin (1:400 dilution in PBS) (Thermo Fisher Scientific Inc. Waltham, United States of America) and nuclei for 3 min with DAPI (1:800 dilution in PBS) (Sigma Aldrich, St. Louis, United States of America).

### 4.7 3D-bioprinting

Bioprinting experiments were conducted on a custom-designed 3D-bioprinting system equipped with modular drop-on-demand printer heads with a microvalve diameter of 300 µm and a valve opening time of 450 µs (Black Drop Bioprinter GmbH, Aachen, Germany). Printer head temperatures were kept the same as for rheological characterizations. The print pressure for the analysis of drop volume, drop area and post-printing cell viability was set according to Table 2.

**TABLE 3:** Mixture ratios for hydrogel blends of agarose, gelatin and collagen I. For experiments containing HepG2 cells, 175 µl DMEM were replaced by a HepG2 stock suspension. Quantities are given in µl.

Material quantity in µl for 1 ml of hydrogel	0.25Ag	0.50Ag	0.25Col	0.25Ag 0.25Col	0.50Ag 0.25Col	0.25Col 3Gel	0.25Ag 0.25Col 3Gel
H20	250		250	250		125	
10x DMEM				75	75	75	75
DMEM	500	500	500	175	175	175	175
10 mg/ml agarose	250	500		250	500		
20 mg/ml agarose							125
80 mg/ml gelatin						375	375
10 mg/ml collagen			250	250	250	250	250

The drop volume was measured with an embedded SmartDrop system (BioFluidix GmbH, Freiburg, Germany) for 500 drops per material, with the number of detected drops per print pressure given in Supplementary Figure S2. The area of printed drops was measured on glass microscopy slides (Marienfeld, Lauda, Germany), PDMS (10:1 mix, SYLGARD™184 Silicone Elastomer, The Dow Chemical Company, Midland, United States of America), and untreated polymer cover slips (ibidi GmbH, Gräfelfing, Germany). 10 drops were printed onto each substrate at print pressures between 0.1 and 0.5 bar and imaged under the microscope.

### 4.8 Calculation of shear stress inside nozzle

As previously described (Blaeser et al., 2016), the shear stress was estimated using the Ostwald–de Waele relationship (Power-Law) in combination with the law of Hagen–Poiseuille. The flow consistency index K and flow behavior index n were determined from the previous shear viscosity measurements. Further factors comprised the nozzle radius $r_{valve}$ , the nozzle opening time $t_{valve}$ , and the drop volume $V_{drop}$ :

$$\bar{\tau }=\frac{1}{2}K∙{\left[\frac{V_{drop}\left(\frac{1}{n}+3\right)}{\pi r_{valve}^{3}∙t_{valve}}\right]}^{n}$$ (1)

### 4.8 Statistical analysis

Statistical significance was determined using one-way ANOVA for gel structure, two-way ANOVA for printability, and three-way ANOVA for cellular behavior and bioprinting, all with *post hoc* Tukey test for a=0.05 . Graphical data shows the mean with error bars as standard error of the mean (SEM), with significance depicted as ns for no significance, * for *p* > 0.05, ** for *p* > 0.01 and *** for *p* < 0.001. Error bars for average nozzle shear stress (Figure 7D) were calculated through error propagation of SEM of variables in Eq. 1.

## Data Availability

The raw data supporting the conclusions of this article will be made available by the authors, without undue reservation.
