# Supplementary material for: Influence of the physico-chemical bioink composition on the printability and cell biological properties in 3D-bioprinting of a liver tumor cell line
Source: Front Bioeng Biotechnol. 2023 Feb 23;11:1093101. doi: 10.3389/fbioe.2023.1093101 (PMC9996333; doi:10.3389/fbioe.2023.1093101)
Supplement: Supplementary file 1 [file DataSheet1.pdf]

Supplementary Material

1 SEM images of agarose, collagen and gelatin blends

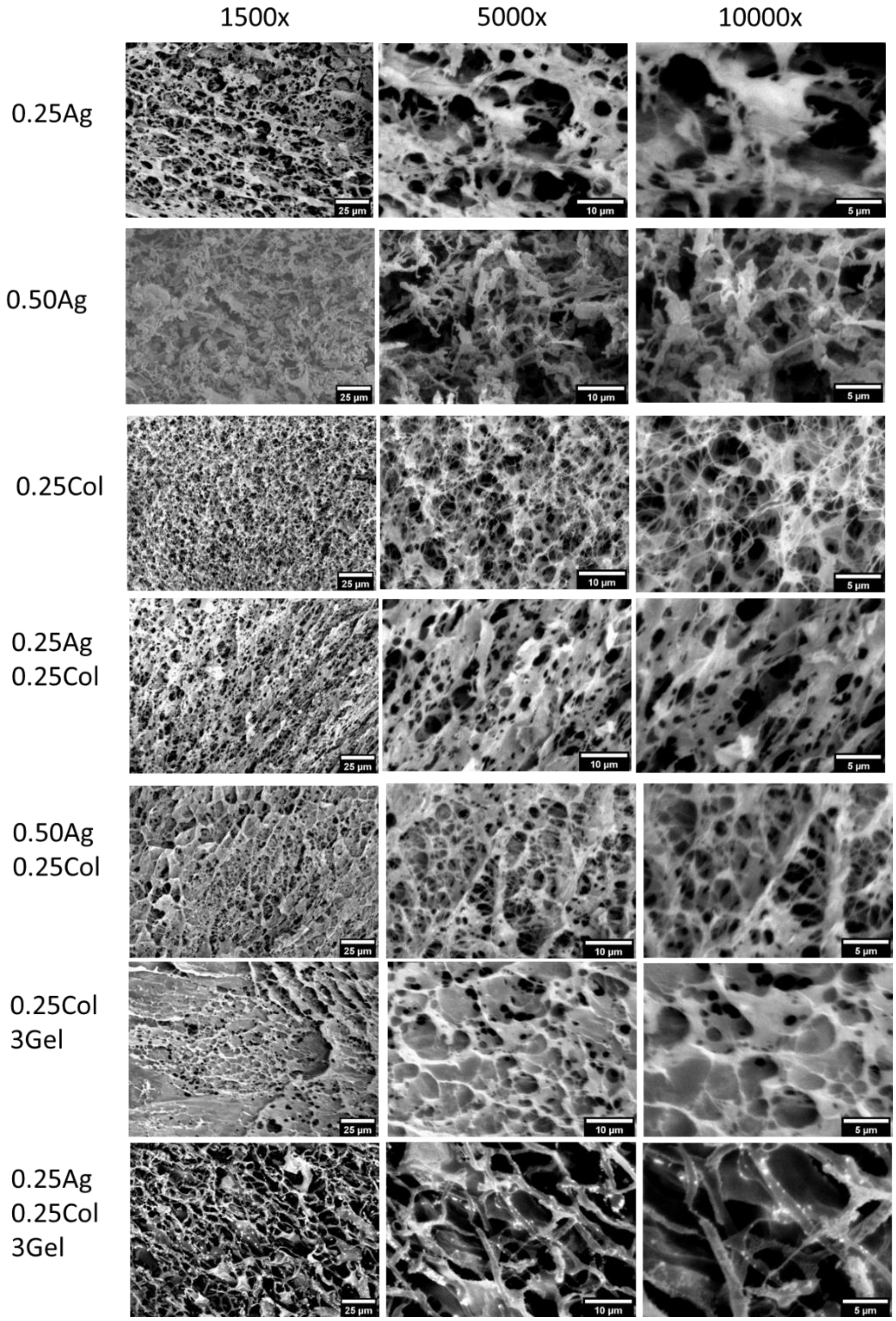

Figure 1: SEM images of freeze-dried and sputtered material blends at 1500x, 5000x and 10000x magnification.

2 Volume of printed drops measured in flight using a "SmartDrop" system

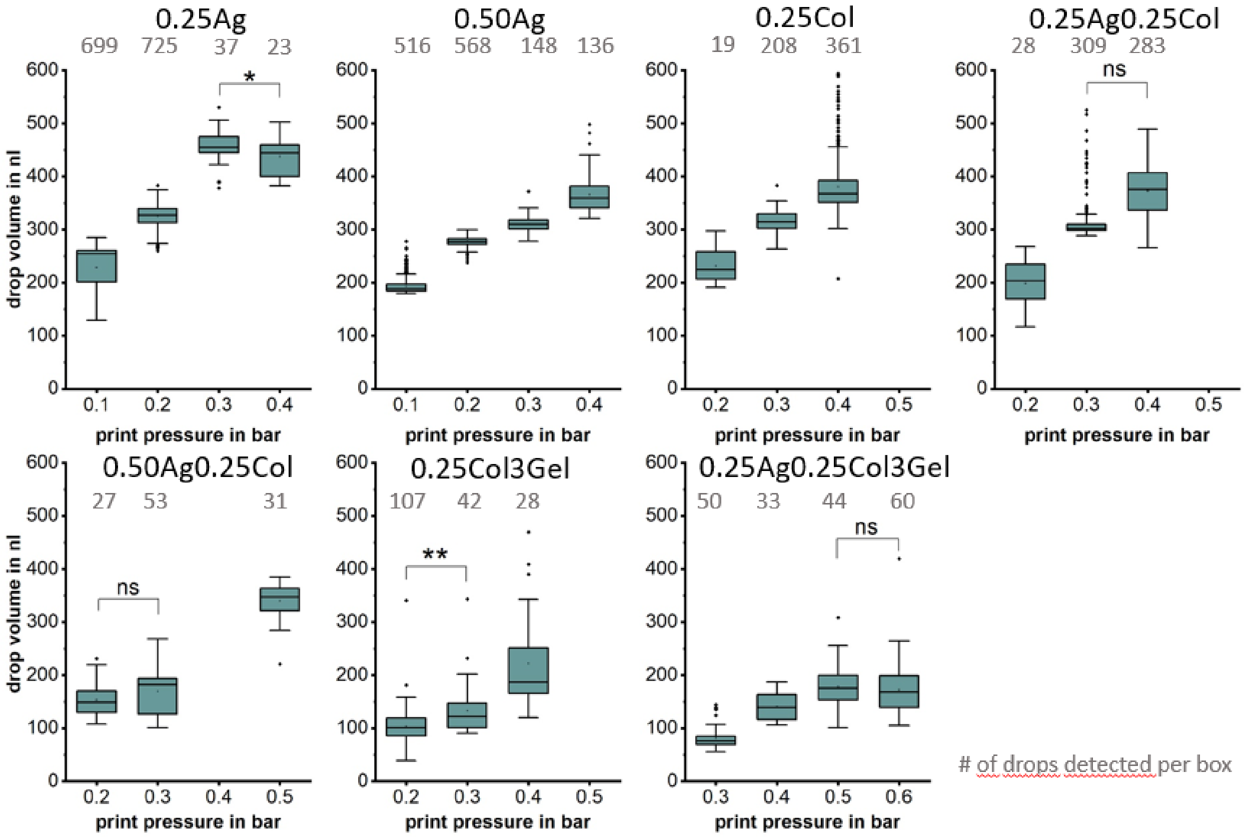

Figure 2: Drop volume for all materials for various print pressures as measured using the "SmartDrop" system. The number of detected drops per measurement are noted on top of the box plots. Significance determination between group mean was done using one-way ANOVA with post hoc Tukey test, with all comparison found to be significant with $p<0.001$ unless stated otherwise (ns = non-significant, * = $p<0.05$, ** = $p<0.01$).

A video of printed drops of 0.50Ag as imaged by the "SmartDrop" system in flight can be found in the Supplementary Material, Video1.

3 Image processing and data treatment for determining cell viability and agglomeration

Fluorescence images obtained were analyzed using a custom macro in ImageJ (NIH, USA)[1] and MATLAB (The MathWorks, Inc., Natick, MA, USA) in order to count the number of live and dead cells and calculate the viability. The images were converted into 16-bit, the background subtracted using a rolling radius of 120 pixels for FITC images and of 60 for Texas Red, and a median filter of radius 3 pixels was applied. To identify the cells or clusters a threshold was defined using the Auto Threshold plug-in using the Intermodes algorithm. The rolling radius and the threshold algorithm was adapted for some images where debris or very large cell agglomerates were not segmented

properly. The Analyze Particles function was used to identify all cells and clusters larger than 4 pixels. The results obtained include the area of each particle, the X and Y coordinates of the centroid and the number of particles found in each image. They were exported in one CSV file per image for further processing using MATLAB. Average cell and nucleus area in pixels were measured on the threshold images using ImageJ (n>50).

Whenever the area of the particle was greater than the average cell or nucleus ± 99% confidence interval, the particle was assumed to be a cluster of cells. The area was divided by the average area of a single cell –or nucleus– previously measured. When the area of the particle was smaller, the particle was considered as noise and not counted. Cell viability per image was calculated as follows:

$$Viability = \frac{n_{green}}{n_{green} + n_{red}} \times 100\% \quad \textbf{Equation 1}$$

With n_{green} the number of green –live– cells counted in the FITC image, and n_{red} the number of red –dead– cells.

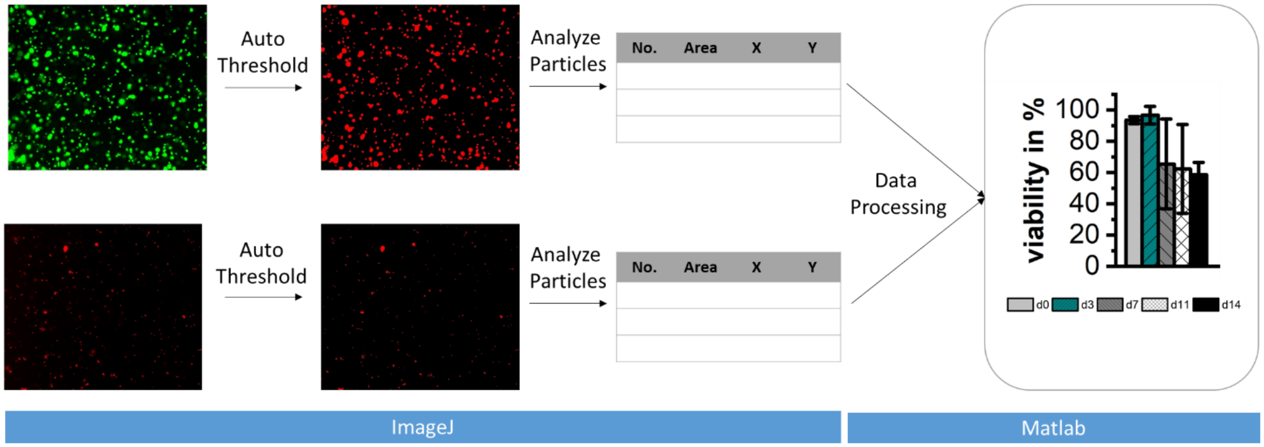

Figure 3: Schematic representation of the image analysis workflow to calculate cell viability using ImageJ and Matlab.

1. Schneider CA, Rasband WS, Eliceiri KW. NIH Image to ImageJ: 25 years of image analysis. *Nat Methods*. 2012;9(7):671-675. doi:10.1038/nmeth.2089

4 Fluorescence microscopy images of HepG2 cells showing morphology in hydrogels

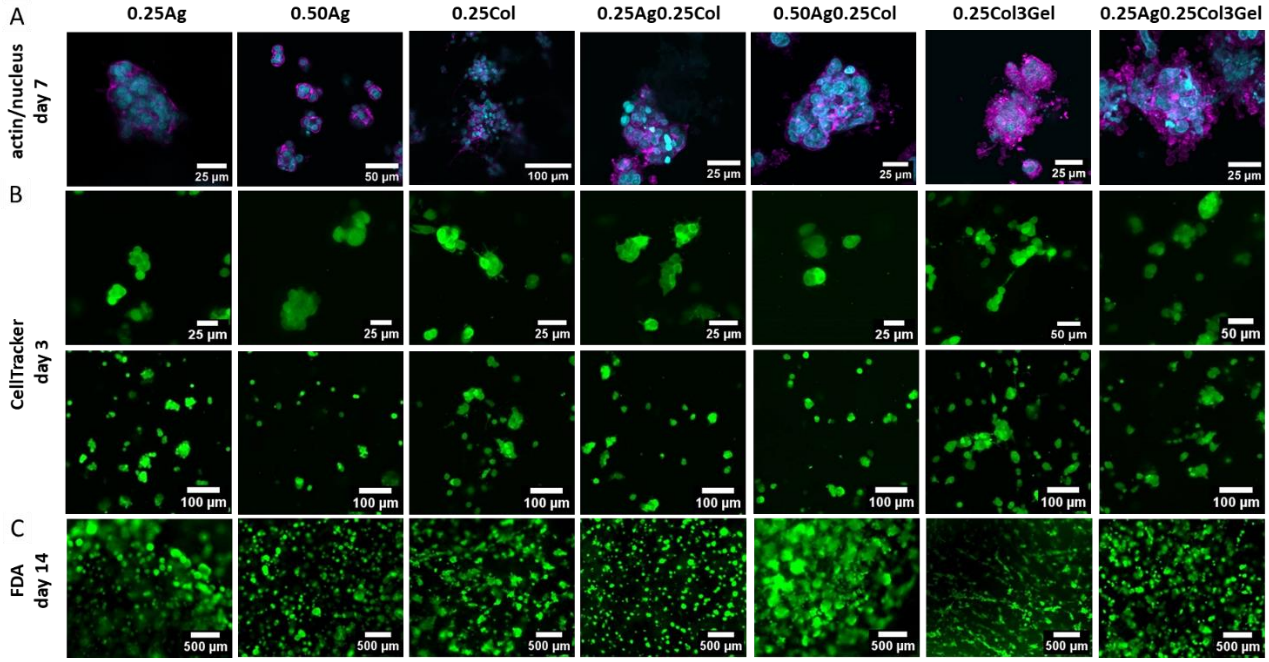

Figure 4: Fluorescence microscopy images of HepG2 cells in all seven materials with actin filaments (magenta) and cell nuclei (blue) stained on day 7 (A), cells cultured with CellTracker green on day 3 (B) and live cells stained with FDA on day 14 (C).

5 Post-printing cell viability of HepG2 cells

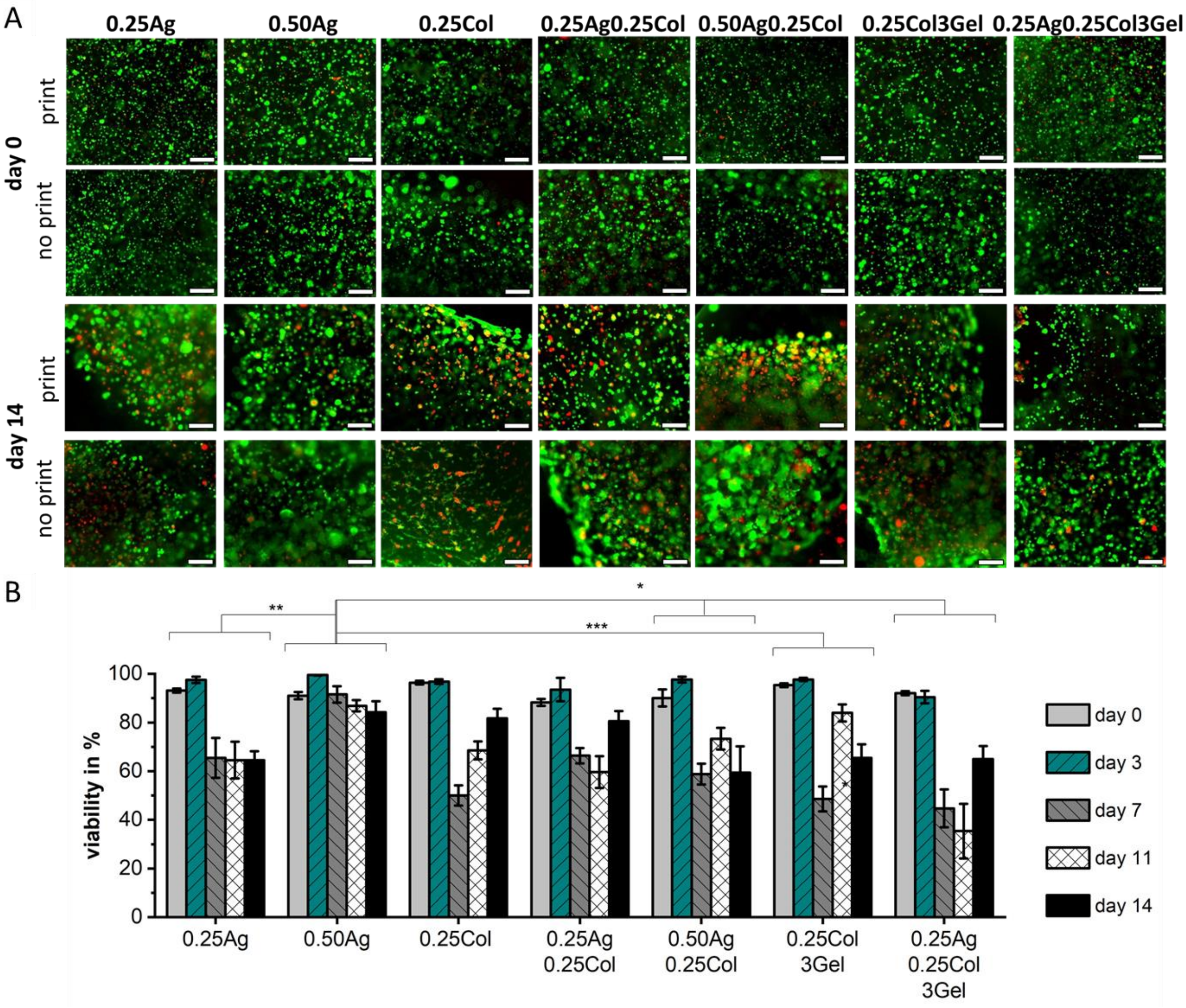

Figure 5: Live (green) and dead (red) staining of HepG2 cells at day 0 and day 14 both after printing and for a no-print control group (A). Images exhibit no distinct difference between printed and no-print control group, with scale bar showing 500 µm. Post-printing viability over 14 days was calculated using ImageJ as explained in S3; n=3 with * for $p<0.05$, ** for $p<0.01$ and *** for $p<0.001$ (B).
